# Restoration management of cattle resting place in mountain grassland

**DOI:** 10.1371/journal.pone.0249445

**Published:** 2021-04-01

**Authors:** Teowdroes Kassahun, Klára Pavlů, Vilem Pavlů, Lenka Pavlů, Jan Novak, Petr Blažek

**Affiliations:** 1 Department of Ecology, Czech University of Life Sciences Prague, Prague, Czech Republic; 2 Department of Weeds and Vegetation of Agroecosystems, Crop Research Institute, Prague, Czech Republic; 3 Faculty of Agrobiology and Food Resources, Slovak University of Agriculture in Nitra, Nitra, Slovakia; 4 Faculty of Science, Department of Botany, University of South Bohemia, České Budějovice, Czechia; 5 Institute of Entomology, Academy of Sciences of the Czech Republic, České Budějovice, Czechia; Feroze Gandhi Degree College, INDIA

## Abstract

This study investigated the effect of restoration management of a weed-infested area, previously used as cattle resting place, on herbage production and nutrient concentrations in the soil and herbage. The experiment was undertaken from 2004 to 2011 at the National Park of Nízké Tatry, Slovakia. Three treatments were applied: (i) cutting twice per year, (ii) herbicide application, followed after three weeks by reseeding with a mixture of vascular plant species and then cut twice per year, and (iii) unmanaged. Treatments had significant effect on biomass production and concentration of nutrients in the soil and in herbage. Nutrient concentrations in herbage and in soil declined progressively under the cutting treatments and reached optimum ranges for dairy cattle at the end of the experiment when herbage N was less than 15 g kg^-1^ and herbage P was 3.4 g kg^-1^. There was also a strong positive relationship under the cutting treatments between soil nutrient concentrations and herbage nutrient concentrations for N, P, K, Mg and Ca. Although the cutting management as well as the combination of herbicide application with cutting management reduced nutrient concentrations in the soil and in herbage, the nutrient concentrations remained relatively high. We can conclude that restoration of grassland covered with weedy species like *Urtica dioica* and *Rumex obtusifolius*, with excessive levels of soil nutrients, cannot be achieved just by cutting and herbicide application.

## Introduction

Grasslands are one of the most important components of the landscape in temperate regions of Europe [1]. Although the development of grasslands, and semi-natural grasslands in particular, is largely related to the history of agricultural management, their existence faces serious threats from either intensification of management or from land abandonment. These threats have increased especially in recent decades [2]. It is widely assumed that when grazing is stopped and abandonment proceeds, a natural succession would take place leading to restoration of the land to its climax state, which is typically dominated by perennials [3]. Unfortunately, this does not happen often and instead it remains dominated by annual species [4] and invasive annual weeds. Persistence of many annual species in grassland is further supported by the increased rate of nutrient turnover, which is facilitated by the invasion of exotic annual species [5]. This challenge is exacerbated in high-altitude grasslands that were previously managed by regular grazing or as resting places for cattle, where they typically receive excessive nutrient returns from cattle excreta.

Restoration of botanical composition of semi-natural grasslands in these situations requires a reduction in the cover of weed species and improved performance of the perennial native species. This requires an integrated approach using multiple techniques, such as mechanical disturbance, fire, and in some cases the use of herbicides [6]. Among the various methods, the use of herbicide has been found to be an effective way to reduce or control weeds in grassland ecosystems, especially when mechanical control is expected to be too damaging [7]. Different types of herbicides are used, sometimes with formulations designed to target specific species such as *Rumex* spp., and others that are non-selective. Glyphosate is one of the most frequently used herbicides in the global market due to its effectiveness, relatively low cost, and its broad-spectrum application [8]. When the objective is to increase native species abundance and richness, broadcast spraying of herbicides is recommended [9,10]. Other studies recommend application of herbicide before the introduction of native species in order to open the sward and thereby increase opportunities for greater seedling density and survival.

Since its introduction in the 1970s, glyphosate remained popular among farmers across the world due to its broad-spectrum weed control capability [11]. During these periods, several countries in Central Europe such as Slovakia were struggling with the challenge of managing invasive weed species. Unfortunately, herbicide was widely used and glyphosate was the chosen chemical. Several studies have been conducted documenting the sever effects glyphosate based herbicide products and its wide spread presence in aquatic and terrestrial environments [12]. Among the main concern regarding glyphosate is its negative effect on non-target plant tissues and unintended areas through process like off target herbicide movement and root uptake [11]. Other consequences of glyphosate include reduction in soil dwelling earthworms reproduction capacity [13], bringing behavioral change in honey bees [14] and affecting the growth of aquatic bacteria and microalgae [15]. When application of herbicide is considered as unsuitable (e.g. due to off-site effects) cutting or mowing is considered [16,17]. Cutting especially has several attributes that can help control weeds. It can arrest flowering of weeds and thereby minimize the production of seeds and breaking their life cycle, leading to their eradication, and it can also increase tillering in some grasses and promote defoliation tolerant species [18–20].

Although the negative effects of non-selective herbicide application is well documented, very little is known about the effects of herbicide application combined with cutting, on changes in the nutrient content in herbage and soil, especially in mountain grasslands that are normally managed by grazing or used as a resting place. When control of invasive plant species is planned, intervention measures or control methods must be assessed not only in terms of their effectiveness in removing targeted species but also their impact on the ecosystem [21]. Herbicides like glyphosate are normally sprayed directly on to growing plants, and never applied intentionally on to the soil. Nevertheless, in open swards especially, there is a high chance that a significant portion may reach the soil surface during application. This technique was widely used in Slovakia, to eradicate invasive species. Against this background, a study was conducted in a mountain grassland area in Slovakia that is covered with weedy species (*Rumex obtusifolius* and *Urtica dioica*). In order to attempt to restore the grassland to its previous status, treatments that included a restoration measure of cutting and of herbicide (glyphosate) application combined with cutting, followed by reseeding with mixed grass species were applied. These treatments were selected based on discussion with administrators and managers of the study site (National Park of Nízké Tatry, Slovakia) and the existing practice of defoliation (cutting) and herbicide application, which was widely used in the country during the study period. However, this approach raised a number of critically important questions that justified the monitoring of the site for 8 years and which are reported in this paper. These questions are: does cutting management, herbicide application, or a combination of both followed by reseeding have an effect on (i) herbage productivity; (ii) nutrient concentrations in herbage and soil, and (iii) how fast are nutrients depleted from the soil.

## Materials and methods

### Study site and experiment design

This study was conducted with approval from the Ministry of Environment of the Slovak Republic.

In 2004, a randomized block experiment was set up at 1140 m a.s.l. in the National Park of Nízké Tatry (48°51.22´N, 19°14.57´E), Slovakia. At the study site, the mean annual precipitation and temperature were 800 mm and 8°C respectively. The snow cover, which is higher than 10 mm, is 160 days per year. The soil type is classified as cambisol, and as the depth of the soil increases the lower the proportion of clay and silt fraction and the higher the proportion of sand fraction. The most dominant species recorded in the experiment plots were *U*. *dioica*, and *R*. *obtusifolius*. The total cover (%) of forbs, grasses, legumes and the mean value of the most abundant species in the experiment site under each treatment for the year 2004 (start of the experiment) and 2011 (end of the experiment) are shown in Table 1.

**Table 1:** Total cover (%) of forbs, grasses, legumes and the cover (%) of the most abundant species in each treatment.

	2004	2011		
Species	Treatment			
	Baseline	U	2CH	2C
*Achillea millefolium*	0±0.00	0±0.00	8±0.57	5±0.57
*Alchemilla vulgaris*	0±0.00	0±0.00	5.25±0.57	3.75±1.15
*Agrostis capillaris*	0±0.00	0±0.00	0.75±0.57	4.5±0.57
*Dactylis glomerata*	1±0.33	0±0.00	3±0.00	1±0.00
*Festuca pratensis*	0±0.00	0±0.00	6.25±1.0	1.5±0.57
*Festuca rubra ssp*. *rubra*	0±0.00	0±0.00	4.5±1.15	1.5±0.57
*Myosotis sylvatica*	4±0.53	4.25±0.00	0±0.00	0±0.00
*Phleum pratense*	0±0.00	0±0.00	10±1.00	0±0.00
*Poa pratensis*	0±0.00	0±0.00	7.5±0.57	0±0.00
*Poa trivialis*	4±1.41	3.75±0.57	0±0.57	13.25±1.00
*Ranunculus repens*	0±0.00	0±0.00	0.5±0.57	9.25±1.00
*Rumex obtusifolius*	76.5±1.20	76±0.57	0±0.00	3±1.00
*Taraxacum officinale* agg.	0±0.00	0±0.00	6.5±0.57	7±1.53
*Trifolium repens*	0±0.00	0±0.00	23±1.15	25.5±1.53
*Trisetum flavescens*	0±0.00	0±0.00	11.25±0.57	5±1.00
*Urtica dioica*	14.5±0.83	15±0.00	0±0.00	0±0.00
**Total cover of grass**	**5±1.27**	**4.75±0.57**	**43.75±2.64**	**27.25±3.78**
**Total cover of legumes**	**0±0.00**	**0±0.00**	**27.5±0.57**	**27±1.73**
**Total cover of forbs**	**95±1.30**	**95.25±0.57**	**28.75±1.53**	**34±3.61**

Numbers represent mean values in unmanaged (U), cutting twice per year (2C) and herbicide application, after three weeks reseeded with grass mixture and cut twice per year (2CH) for the year 2004 and 2011. ± Value indicate Standard deviation (S.D.).

The experimental site was previously used for grazing and then for herding of heifers. However, during the decade before 2004, it was abandoned without any grazing or cutting management. The experiment was arranged in four randomized blocks each with the following treatments: (i) Unmanaged (U), (ii) Cutting twice per year (2C), and (iii) Herbicide application and, after three weeks, it was reseeded with 18 mixture of vascular plant species (list of species see Table 2) and subsequently cut twice per year (2CH). Glyphosate (active substance IPA 480 g.l.; Roundup; Monsanto) herbicide was applied on to the leaves of plants at 3 l ha^-1^ (0.30 ml agent + 20 ml water on 1 m^2^) with a sprayer in the spring of 2004. Altogether 12 (three treatments x four blocks) plots were established for the experiment with each plot measuring 15 m^2^.

**Table 2:** List of vascular plant species that were reseeded after application of herbicide on the 2CH treatment (herbicide application, then after three weeks reseeded with grass mixture and cut twice per year).

Species	Proportion of the mixture (%)
*Dactylis glomerata* L.	25.00
*Festuca pratensis* Huds.	10.00
*Phleum pratense* L.	10.00
*Poa pratensis* L.	10.00
*Festuca rubra* L.	5.00
*Trisetum flavescens* (L.) P Beauv.	5.00
*Trifolium repens* L.	15.00
*Trifolium pratense* L.	3.00
*Lotus corniculatus* L.	3.00
*Plantago lanceolata L*.	2.00
*Achillea millefolium* L.	2.00
*Carum carvi* L.	2.00
*Taraxacum officinale* Weber	2.00
*Alchemilla vulgaris* L.	2.00
*Daucus carota* L.	1.00
*Acetosa pratensis Mill*.	1.00
*Leucanthemum vulgare* Lam.	1.00
*Prunella vulgaris* L.	1.00

### Herbage biomass production and herbage chemical properties

The above ground dry matter (DM) biomass production for the whole vegetation season was determined in each of the years 2005–2011. It was calculated as the sum of sampled DM biomass (harvested in the spring and autumn for 2C and 2CH treatments). The harvested biomass in each treatment was measured in sub plots each of 6 x 2.5 m within each of the 15 m^2^ experimental plots. In each treatment plot, the above ground biomass was cut 3 cm above the ground. In order to avoid any residual effect of herbage collection from previous years, the sampling for the U treatment was conducted from different sub plots outside the designated experimental plots in each year. To determine the DM content of biomass, and thus the DM yield, the harvested herbage samples were weighed fresh, and oven dried at 80°C.

Concentrations of N, P, K, Mg and Ca were determined from the herbage samples collected in autumn for the DM biomass determinations. The samples were used for analysis, after digestion in aqua regia by ICP-OES. The crude fibre was determined using Weende analysis [22].

### Soil chemical properties

Every autumn (in September) after the last round of cutting, soil samples (consisting of three sub samples) were randomly collected from depths of 0–10 cm and 10–20 cm of the soil profile using an auger, from each of the 15 m^2^ treatment plots for the years 2004 to 2011. The soil samples were oven dried at 100 ^o^C, ground in a mortar, and sieved to 2 mm after removal of biomass residues and living roots. Soil pH was determined in potassium chloride solutions. Plant-available P, K, Mg, Ca were extracted by Mehlich III reagent [23]. Total Nitrogen (N_tot_) was determined using the Kjeldahl method and soil organic carbon (C_org_) using the oxidimetric method according to Tiurin.

### Statistical analysis

A general linear model (GLM) with treatment as fixed effects, replication as a random effect and year as continuous predictor was used to identify the effect of year, treatment and the year x treatment interaction, on nutrient concentrations in the herbage and in the soil for the whole experiment period. One-way ANOVA followed by Tukey HSD test was used to identify significant differences between treatments for chemical properties of soil and herbage for the last year of the experiment (2011). In order to control for false-discovery rate (FDR), we applied Benjamini-Hochberg’s procedure [24]. All univariate analyses were performed using Statistica 13.1 [25].

To illustrate the influence of treatments on nutrient concentration of the soil and the herbage over the entire experiment period, a partial principal component analysis (pPCA) with replication as covariate was conducted. Canoco 5 was used to perform pPCA [26]. Moreover, to identify the relationship between plant available nutrients in the soil and the nutrient contents in the herbage a linear regression analysis was applied.

## Results

### Herbage biomass production

**Fig 1:**
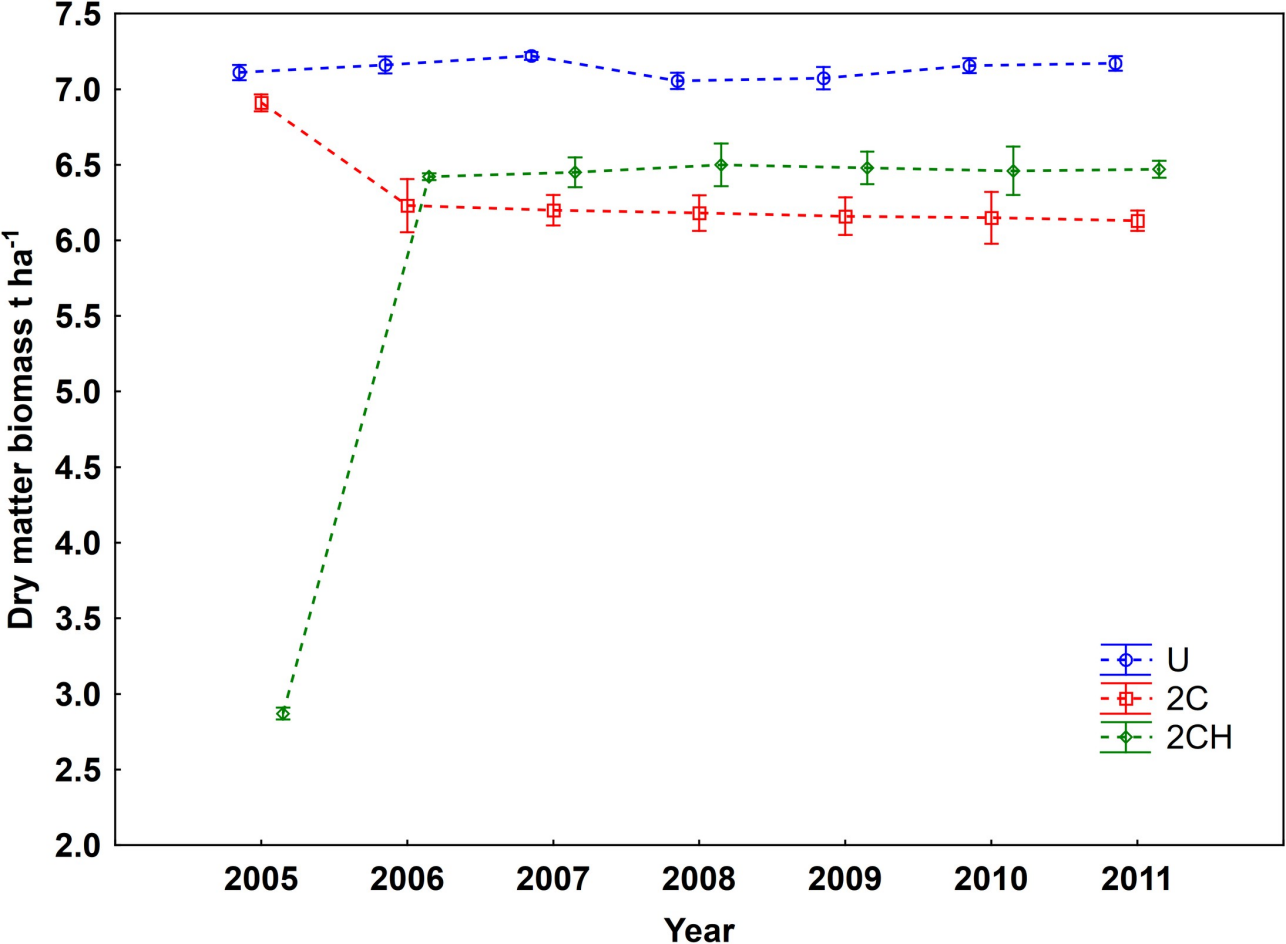
Dry matter biomass production in investigated treatments over the years 2005–2011. Error bars represent standard error of the mean (SE). For treatment abbreviation (U, 2C, 2CH) see Table 1.

As anticipated, the data on DM biomass showed considerable annual variation especially during the early stages of the experiment. The response of biomass production to treatments resulted in statistically significant differences between U, 2C, and 2CH treatments. The GLM analysis showed that DM biomass was significantly affected by year and treatment (*P*<0.001) as well as the interaction of year x treatment (*P*<0.001) (Table 3). From 2005 to 2011, the mean annual values of herbage biomass production were as follows: 7.1 t ha^-1^ (U), 6.3 t ha^-1^ (2C) and 5.9 t ha^-1^ (2CH). Total DM biomass remained above 7 t ha^-1^ under the U treatment and remained stable during the entire experiment period, while under 2C treatment it slowly but continuously declined from approximately 7 to 6 t ha^-1^ (Fig 1). A large increase in DM biomass was observed under the 2CH treatment, from 2.5 to 6.5 t ha^-1^ at the beginning of the experiment, and it then stabilized at 6.3 t ha^-1^ (Fig 1). During the 7 years of biomass sampling, DM biomass production was significantly higher and stable under U, but after 2 years of the experiment, the DM under the cut treatments (2C and 2 CH) also became stable (Fig 1).

**Table 3:** Result of GLM analysis (year, treatment, year x treatment) of herbage and soil chemical properties for the whole experiment period.

		Year		Treatment		Year x Treatment	
		*F-*ratio	*P*-value	*F*-ratio	*P*-value	*F*-ratio	*P*-value
**Herbage**	DM (%)	8.23	**0.005**	29.36	**<0.001**	17.88	**<0.001**
	Crude Fibre	6.50	**0.013**	2.92	0.060	2.63	0.078
	N	253.67	**<0.001**	0.24	0.781	64.73	**<0.001**
	P	326.79	**<0.001**	17.33	**<0.001**	80.33	**<0.001**
	K	292.26	**<0.001**	0.08	0.923	71.54	**<0.001**
	Mg	31.13	**<0.001**	21.48	**<0.001**	8.12	**<0.001**
	Ca	51.63	**<0.001**	3.59	**0.032**	12.40	**<0.001**
**Soil**							
	N_tot_	178.29	**<0.001**	0.31	0.737	49.01	**<0.001**
	P	76.99	**<0.001**	4.59	**0.013**	19.19	**<0.001**
	K	171.17	**<0.001**	1.16	0.318	49.12	**<0.001**
	Mg	67.08	**<0.001**	0.22	0.805	18.12	**<0.001**
	Ca	27.28	**<0.001**	1.71	0.181	3.53	**0.034**
	C_org_	10.96	**<0.001**	0.02	0.980	3.92	**0.023**
	C: N	204.17	**<0.001**	1.81	0.170	48.12	**<0.001**
	pH/KCl	15.51	**<0.001**	5.08	**0.008**	3.49	**0.034**

*F* represents the value derived from *F* statistics in GLM and *P* represents the resulting probability value. Significant results (after table-wise Benjamini-Hochberg’s FDR correction) are highlighted in bold.

### Herbage chemical properties

**Fig 2:**
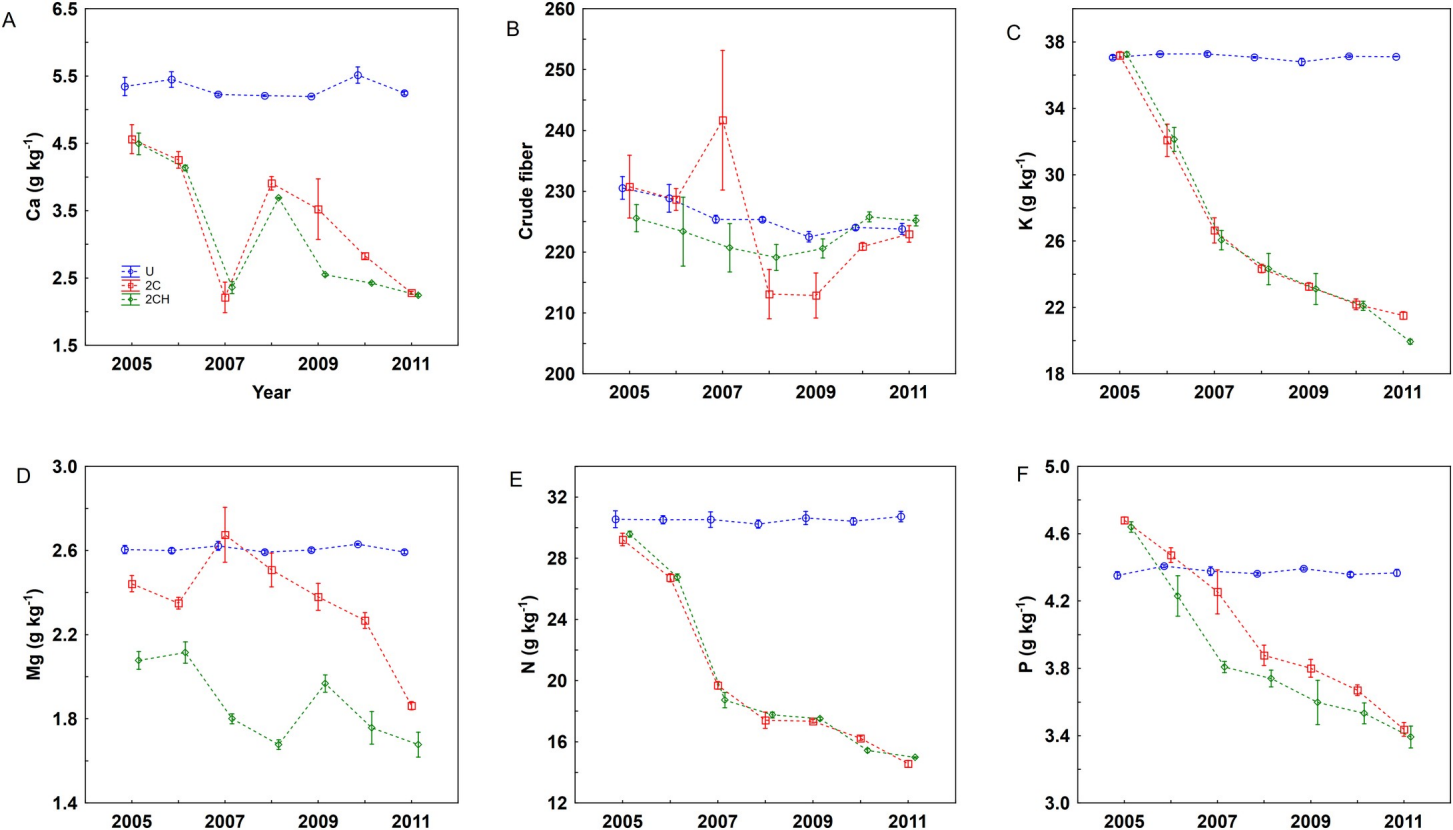
Concentration of Ca (A), Crude fiber (B), K (C), Mg (D), N (E) and P (F) in the herbage. Error bars represent standard error of the means (SE). For treatment abbreviation (U, 2C, 2CH) see Table 1.

The GLM analysis revealed a significant effect of treatment on herbage nutrient concentrations of P, Mg and Ca, but not on crude fiber (CF), N and K. However, a significant effect of the year, and the interaction of year x treatment, was recorded for all nutrient concentrations except CF (Table 3; Fig 2). The results of one-way ANOVA showed that treatment had an effect on all herbage chemical properties except on CF (Table 4).

**Table 4:** Mean soil and herbage characteristics and mean dry matter biomass under the different treatments in 2011.

Characteristics	U	2C	2CH	*F-* ratio	*P-* value
**Herbage nutrient**					
CF g kg^-1^	223.82±0.89	222.99±1.35	225.18±0.86	1.11	0.38
N g kg^-1^	30.73±0.34 a	14.56±0.22 b	14.97±0.04 b	1501.01	**<0.001**
P g kg^-1^	4.37±0.021 a	3.44±0.04 b	3.39±0.07 b	143.44	**<0.001**
K g kg^-1^	37.12±0.02 a	21.51±0.23 b	19.94±0.16 c	3458.48	**<0.001**
Mg g kg^-1^	2.59±0.01 a	1.86±0.02 b	1.67±0.06 c	181.49	**<0.001**
Ca g kg^-1^	5.24±0.03 a	2.28±0.02 b	2.24±0.20 b	5647.75	**<0.001**
**Soil Chemical Properties**					
N_tot_ mg kg^-1^	6825.01±128.41 a	3007.50±170.41 c	4075.11±155.91 b	166.63	**<0.001**
P mg kg^-1^	400.01±7.07 a	75.04±2.88 b	135.00±26.29 b	119.62	**<0.001**
K mg kg^-1^	920.11±1.66 a	267.50±12.50 b	250.10±18.25 b	893.58	**<0.001**
Mg mg kg^-1^	455.03±17.08 a	197.50±7.50 b	222.51±19.31 b	83.92	**<0.001**
Ca mg kg^-1^	2512.50±26.57 a	1455.01±79.74 b	2115.11±215.27 a	16.02	**<0.001**
C_org_	60810.01±1057.88 a	50220.11±2616.81 b	66047.51±1573.98 a	18.67	**<0.001**
C: N	8.91±0.04 b	16.71±0.11 a	16.23±0.24 a	784.46	**<0.001**
pH/KCl	4.83±0.01 a	4.59±0.03 b	4.55±0.03 b	30.21	**<0.001**

*F*-ratio = *F*-statistics for the test of a particular analysis, *P*-value = corresponding probability value, d.f = (2, 9) in all tests. The numbers reflect the average of four replicates, ± standard error of the mean (SE). Significant results (after table-wise Benjamini-Hochberg’s FDR correction) were highlighted in bold. Significant differences between treatments in Tukey test are indicated by different lower-case letters (alphabetic order represents decreasing values of means, i.e. a represents the largest mean). For treatment abbreviation (U, 2C, 2CH) see Table 1.

The mean concentration of N in herbage dry matter ranged from 14.56 g kg^-1^ (2C) to 30.73 g kg^-1^ (U) and the mean concentration of P ranged from 3.39 g kg^-1^ (2CH) to 4.37 g kg^-1^ (U). Similarly, the lowest concentrations of Mg and K were under treatment 2CH and the highest under treatment U, and ranged from 1.67 g kg^-1^ to 2.59 g kg^-1^ and 19.94 g kg^-1^ to 37.12 g kg^-1^, respectively. The mean concentration of Ca ranged from 2.24 g kg^-1^ (2CH) to 5.24 g kg^-1^ (U) (Table 4).

During the course of the experiment, significant amounts of nutrients were removed in harvested herbage under the cutting treatments. The removal of nutrients at the beginning of the experiment was much greater than in the last year of sampling. For instance, 135 kg ha^-1^ of N, 21.59 kg ha^-1^ of P and 171.31 kg ha^-1^ of K were removed under the 2C treatment at the start of the experiment. In contrast only 60.15 kg ha^-1^ of N, 14.09 kg ha^-1^ of P and 87 kg ha^-1^ of K were removed under 2C in the last year of the experiment (Table 5). Under the 2CH treatment the amount of nutrient concentrations removed in the first year was the lowest compared to the other sampling years. This is consistent with the amount of herbage biomass produced in the same period, which was also low as the treatment was reseeded with grass mixture during that period.

**Table 5:** Amount of nutrients removed in the harvested biomass for the years 2005 to 2011.

Year	Treatment	Nutrients				
		N (kg ha^-1^)	P (kg ha^-1^)	K (kg ha^-1^)	Mg (kg ha^-1^)	Ca (kg ha^-1^)
2005	2C	135.97	21.59	171.31	11.251	21.30
	2CH	56.67	8.93	71.27	3.97	8.47
2006	2C	110.89	18.511	133.35	9.75	17.46
	2CH	114.83	17.92	137.47	9.05	17.82
2007	2C	82.13	17.654	112.02	11.05	9.49
	2CH	81.65	16.34	110.25	7.73	10.18
2008	2C	71.79	16.02	100.26	10.34	16.08
	2CH	77.30	16.29	105.53	7.26	15.98
2009	2C	71.13	15.57	95.49	9.77	14.48
	2CH	75.92	15.66	100.03	8.50	11.06
2010	2C	66.56	15.02	91.00	9.29	11.57
	2CH	66.58	15.24	95.17	7.58	10.48
2011	2C	60.15	14.09	87.89	7.61	9.28
	2CH	64.59	14.71	86.01	7.24	9.71
Total	2C	598.65	118.48	789.58	69.08	99.68
	2CH	537.59	105.12	707.53	51.36	83.70

Numbers represent average of four replicates. For treatment abbreviation (2C and 2CH) see Table 1.

### Soil chemical properties

**Fig 3:**
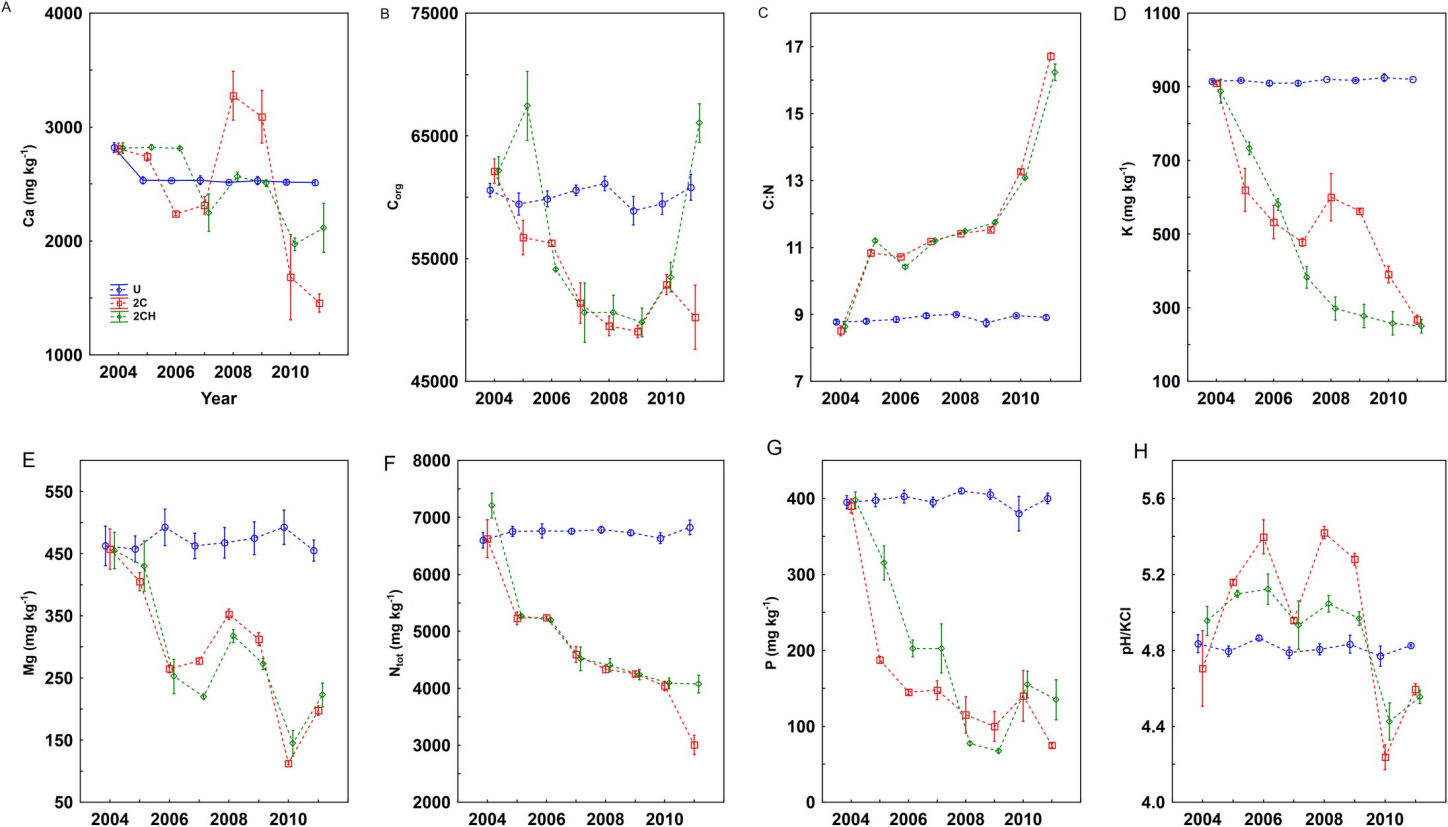
Concentrations of Ca (A), C_org_ (B), C: N (C), K (D), Mg (E), Total N (F), P (G) and pH/KCl (H), in the soil (0–10 cm). Error bars represent standard error of the means (SE). For treatment abbreviation (U, 2C, 2CH) see Table 1.

Concentrations of N_tot_, C_org_, the plant available nutrients K, Mg and Ca, and the C: N in the soil were not significantly affected by treatments. However, year and the interaction of year x treatment, showed significant effects on all concentrations (Table 3; Fig 3). The one-way ANOVA result showed treatment had a significant effect on the soil chemical properties at the end of the experiment (Table 4). The mean concentrations of N, P, K, Mg and pH/KCL were lowest under the cut treatments (2C and 2CH) and the highest under U treatment, and ranged from 3007.50 mg kg^-1^ to 6825 mg kg^-1^, 75.04 mg kg^-1^ to 400.01 mg kg^-1^, 250.10 mg kg^-1^ to 920.11 mg kg^-1^, 197.50 mg kg^-1^ to 455.03 mg kg^-1^ and from 4.55 to 4.83 respectively. The C_org_ and the C:N ratio ranged from 50 220.11 (2C) to 60 810.01 (U) and 8.91 (U) to 16.71 (2C) respectively. The mean concentration of Ca ranged from 1455 mg kg^-1^ (2CH) to 2512 mg kg^-1^ (U) (Table 4).

### Soil and herbage chemical properties

**Fig 4:**
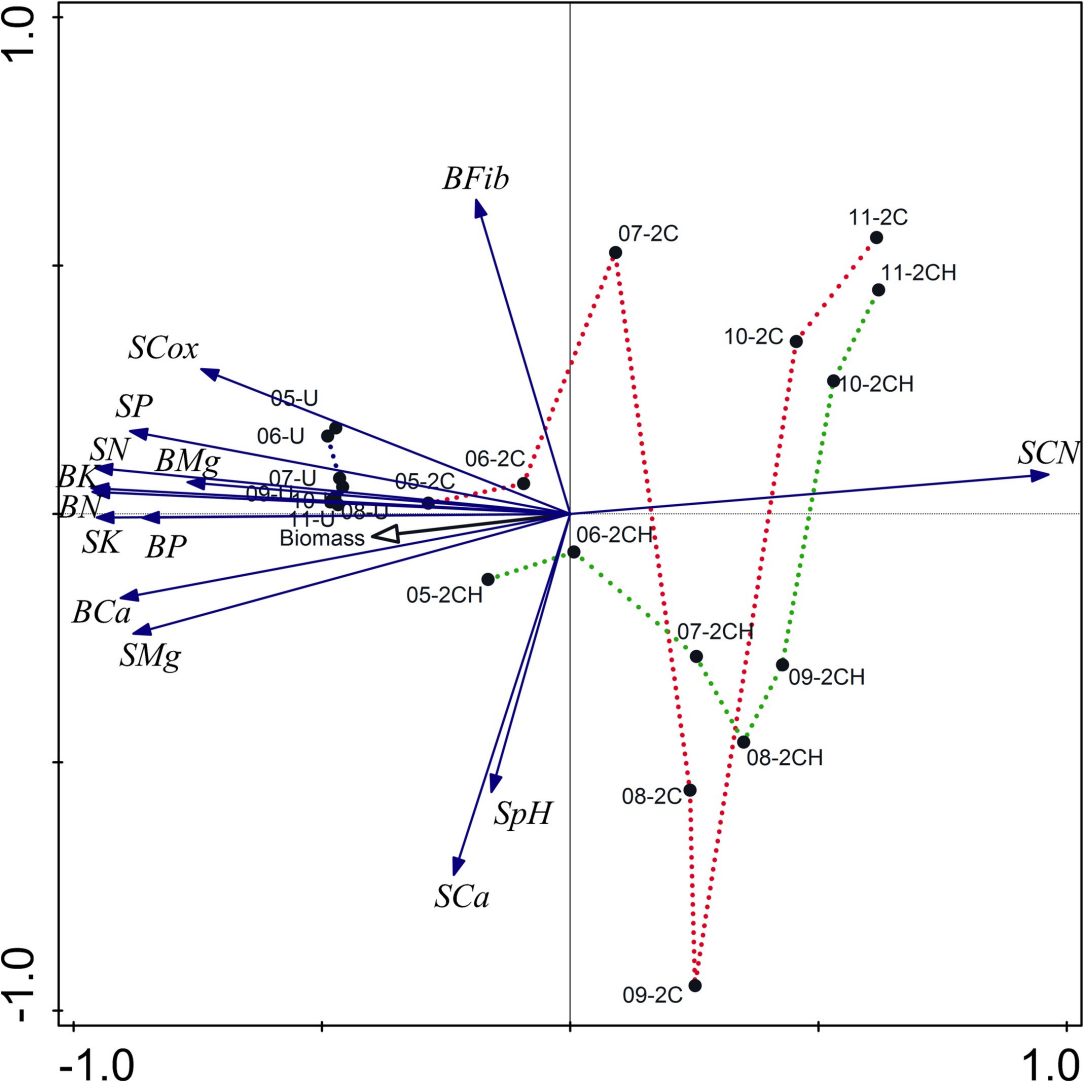
Principal component analysis (pPCA) of the nutrient concentrations in the herbage and in the soil indicating the influence of treatment and its development over the years from 2005 to 2011. The first and the second axis explain 64% and 10%, respectively. Labels include nutrient names and abbreviations: B—herbage nutrient, S—soil nutrient, Fib–crude fibre. Sample labels include treatment abbreviations (see Table 1) and year of sampling.

The pPCA analysis displayed the development and the decline of nutrient concentrations in the soil as well as in the herbage through the course of the experiment. The ordination showed nutrients under U treatment stable throughout the experiment period. In contrast, nutrient concentrations in the herbage and in the soil under the cutting treatments (2C and 2CH) declined starting from the second year, representing 64% of variation for the first axis. There were also small fluctuations in C:N and Ca in the soil as well as pH, representing about 10% of variation in the second axis (Fig 4).

**Fig 5:**
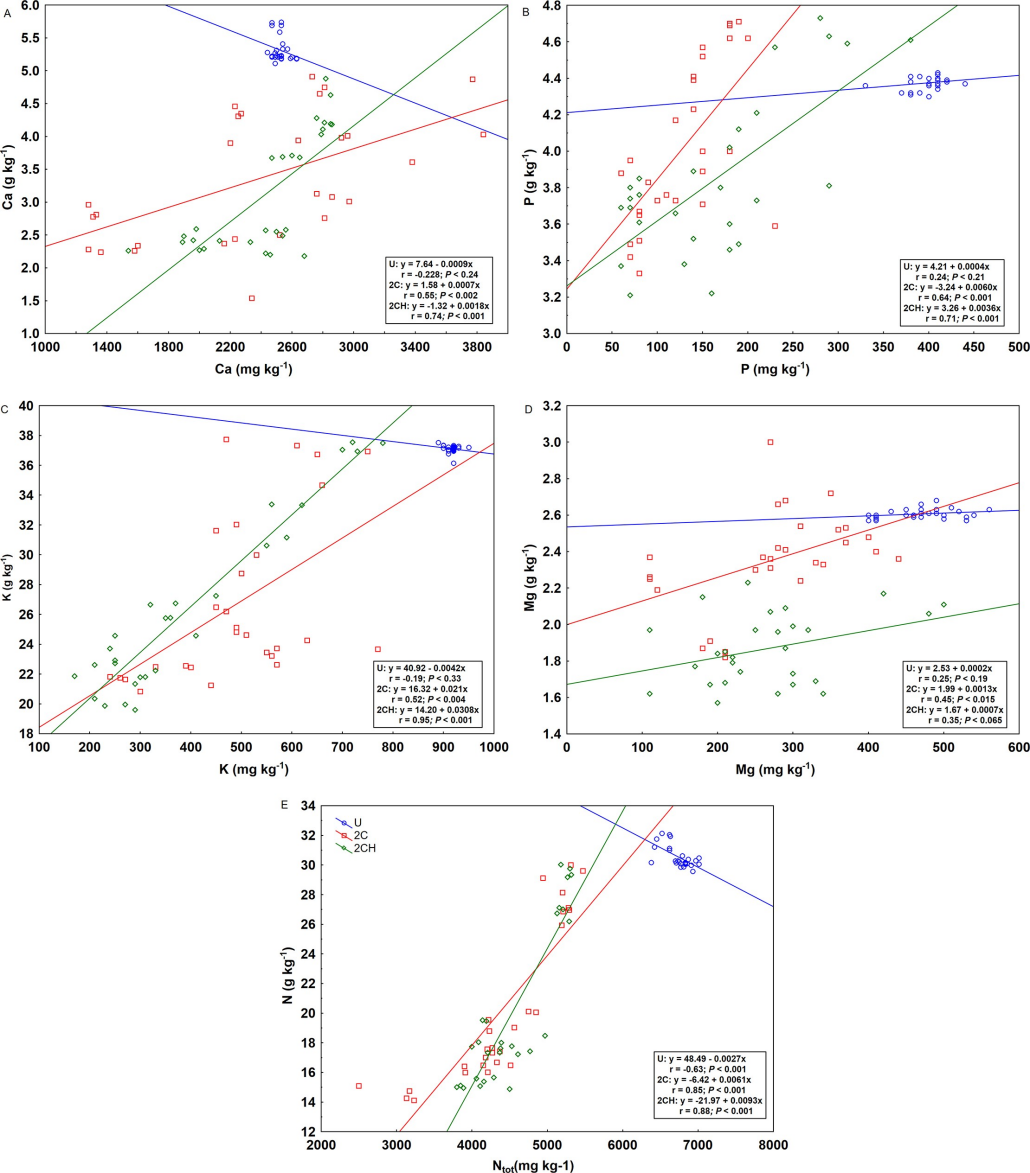
Relationship between concentrations of calcium (A), phosphorus (B), potassium (C), magnesium (D) and nitrogen (E) in the herbage and in the soil. For treatment abbreviation (U, 2C, 2CH) see Table 1.

In the cutting (2C and 2CH) treatments, the concentrations of N, P, K, Mg and Ca in the herbage increased with increasing concentrations of plant available N, P, K, Mg and Ca (Fig 5). Under U treatment, the concentrations of Ca, K and N in the herbage was negatively related to the concentrations of plant available Ca, K and N (Fig 5A, 5C and 5E). In contrast, the concentrations of P and Mg in the U treatment were related positively, and similar to the cutting treatments (Fig 5B and 5D).

## Discussion

### Herbage biomass production

Based on the results from the studied site, we could classify the site as a productive grassland with herbage productivity ranging from 6 to 7.4 t ha^-1^ per year, which is very high for Central European conditions that normally exhibit only 2 to 4 t ha^-1^ per year [27]. Even though we observed a decline in nutrients (discussed later) resulting from the removal of biomass from cutting, the site still produced a high amount of herbage dry matter for Central European conditions. This may indicate a high nutrient reserve within the soil. The variation in the DM biomass production observed during the early period of the experiment could be attributed to climatic conditions such as temperature and precipitation distribution during the vegetation season, as well as the species composition, management applied and altitude [28,29]. Such variability in biomass production is expected and similar results have been reported in other long-term studies in Central Europe [30–32]. One major outcome from this study is that biomass production did not increase either in response to the cutting or to the combination of cutting and herbicide application. Rather it continued to slowly decline and it stabilized throughout the experiment period under the cutting treatments (2C and 2CH). The sharp rise in biomass production at the early stage of the experiment under 2CH treatment is most likely due to the effect of reseeding, which was done at the start of the experiment. Furthermore, the continued decline of N_tot_ and of plant available P and K in the soil (discussed later) also showed similar patterns of decline under the 2C and 2CH treatments. This could be one of the reasons for the continuous decline in biomass production under the cutting treatments. However, the decline in biomass production under cutting management over the duration of the experiment were not huge. This may indicate a relatively high content of N_tot,_ and of plant available P and K in the soil, especially at the start of the experiment.

### Herbage chemical properties

The concentration of P in the herbage declined and reached 3.39 g kg^-1^ under the 2CH treatment at the end of the experiment, whereas at the beginning of the experiment there was a very high concentration of P of around 4.7 g kg^-1^, indicating that biomass growth was not limited by P [33] A relatively high herbage P concentration recorded in the early periods of the experiment could be explained by the high presence of weedy *U*. *dioica*, in the harvested biomass, which is typically characterized by high concentrations of P [34]. The high concentration of P recorded even under the U treatment is quite remarkable when compared to the low concentration (less than 2 g kg^-1^) recorded in low productive semi-natural grasslands [35,36]. Similarly, the high concentrations of K, N and Ca in the herbage, especially during the early periods of the experiment, in all treatments (though much more and stable under U), but declining under 2C and 2CH, could also be attributed to the dominant presence of *U*. *dioica* and *R*. *obtusifolius*, in the harvested biomass as these weed species are considered to have high concentrations of P, N and Ca [34,37–39]. The high nutrient concentrations recorded under the unmanaged treatments is very much connected to the high production of *U*. *dioica* compared with other grassland species. Hence, a higher nutrient concentration is recorded on the above ground biomass under unmanaged treatments throughout the experiment period [40]. On the other hand, the cutting (2C and 2CH) treatments had lower nutrients, which may be explained by the consistent and continuous removal of nutrients that occurs under cutting (Table 5).

At the start of the experiment the relative high proportion of forbs, which were mostly represented by *U*. *dioica*, and *R*. *obtusifolius* in the unmanaged treatment (Table 1) are largely responsible for the high concentrations of nutrients in the herbage. It is common for certain plant functional groups to dominate a grassland after cessation of grazing, and the functional groups are dominated by species that are best suited to the given habitat [41]. In contrast, after the introduction of management (2C and 2CH), it was possible to see that in the final year of the experiment (2011) a significant increase in the cover of graminoids (Table 1) which have relatively lower mineral concentrations than forbs [42,43]. This shift from forbs to graminoids could explain the decline in herbage nutrient concentrations in the 2C and 2CH treatments. According to [44], the optimal concentrations of P and N in the herbage for dairy cattle ranges from 2.3 to 3.7 g kg^-1^ and 19.2 to 25.6 g kg^-1^ respectively. In this study, the optimal values or ranges under the cutting management were reached relatively rapidly in the last years of the experiment.

### Soil chemical properties

Similar to the changes in nutrient concentrations in herbage, the major plant available nutrients N, P, K and Mg in soil on the experiment site showed a decline over the duration of the study under the cutting treatments (2C and 2CH). Although the amount of nutrients that are removed via harvested biomass each year is relatively small [45], it is well documented that cutting with biomass removal over a sustained period can result in nutrient depletion from the soil in the absence of any compensatory fertilizer application [46,47]. The decline for all plant available nutrients in the 0–10 cm soil layer was very similar to the decline recorded for all plant available nutrients in the 10–20 cm soil layers (S1 Fig). For instance, the decline in concentration of P is consistent with a reported decline in concentration of plant available P in a long-term cutting management without application of P and K fertilizer [48]. Similarly, plant available K concentration was expected to decrease under the cutting treatments, as this has been reported in other studies [48,49]. It is generally possible to remove K from the soil quickly by cutting and removing herbage, but similar rapid removal of P is less likely [50]. This result also indicates a positive relationship between the concentrations of herbage P and K and plant available concentrations of P and K (discussed later), which was also confirmed in another study in the Czech Republic [40]. Not surprisingly, the nutrient concentrations in the soil under the U treatment remained largely stable throughout the experiment period. This could be explained by the absence of management and thus no removal of herbage, which would otherwise have led to removal of nutrients similar to that of the plots with cutting treatments.

The removal of Ca and Mg in the soil under the cutting treatments was relatively small. This might be explained by the limited duration of the experiment, which was conducted for only 8 years, as significant removal of such nutrients is likely to require a long-term period [46,48,51]. Concerning the use of the herbicide glyphosate, it contains C, N, and P and these are essential nutrients for soil microorganisms, and the microorganisms acquire C and N by decomposing plant residues and other organic material added to the soil. The ratio of C:N in glyphosate is 3:1 (considered as low) and this may definitely have an immediate impact on soil microbial activity [52]. In our study the C:N ratio under the 2CH treatment showed increases every year. This may indicate that glyphosate application made a contribution to the increased rate of C and N mineralization [53] on the experiment site.

### Soil and herbage chemical properties

Despite the variation in the different axes, the patterns illustrated by the pPCA largely overlapped with the GLM results and, after two years of the experiment, concentrations of most nutrients in the soil, as well as in the herbage, declined sharply except under the unmanaged plots. Even though we can see decline in the nutrient concentrations, they remain high in terms of requirements for grassland species in all treatments. This is perhaps because the area was previously used over a long period (since the 15^th^ century firstly as resting place for sheep and then for heifer) as a resting place for heifers, which would have resulted in excessive amounts of nutrient deposition through urine and faeces on the site. Furthermore, the sharp decline in nutrient concentrations at the early stage of the experiment, which has not been commonly observed in other experiments, can be explained by the high initial amounts of available nutrients in the area as well as the dominance of some nutrient-rich species like *U*. *dioica* and *R*. *obtusifolius*.

The nutrient concentration analyses of P, K, N, Mg and Ca in the herbage and in the soil revealed that the cutting management with biomass removal had an effect on nutrient concentrations in both the soil and in herbage. This could be one of the reasons for the strong positive correlation shown (2C and 2CH) between the herbage and plant available concentrations of P, K, N and Ca. This finding is consistent with the conclusions of previous work [40,50,54], that found P and K showing strong relationships between the soil and herbage concentrations. However, the positive relationship between total soil N content and the concentration of N in the herbage under the cutting management in the current study was contrary to the findings of [50] that showed a negative relationship indicating high total N content in the soil, which means poor soil quality and slow mineralization. The current study was conducted on a site that was used previously as a resting place for cattle, unlike the other studies such as [50], which was a cutting experiment without cattle. Due to the presence of cattle and the site being used as a resting place, high amounts of nutrients through deposition of dung and urine on the site are to be expected. According to [55] the amount of nutrients supplied from dung on an individual patch are 40–60 g N/m^2^, 14–20 g P/m^2^, 16–25 g K/m^2^, 40–60 g Ca/m^2^ and 10–14 g Mg/m^2^. Hence, dung deposition has a significant effect on the chemical status of the soil and thus presents a potential source of available nutrients for plants [56,57]. Furthermore, urine is another source of nutrient especially N, which occurs primarily as a hydrolyzed urea, and is easily plant-available after deposition [58] and enables increased plant biomass N uptake and biomass productivity [59,60].

## Conclusions

1. The introduction of cutting management as well as a combination of cutting with herbicide application and reseeding had effects on herbage production and nutrient concentration in the herbage as well as in the soil.
2. The optimum range of nutrient concentrations in the forage (N and P) which is suitable for dairy cattle were reached within 8 years with low frequency of cutting management.
3. Even though the decline of nutrients from the soil associated with biomass removal was relatively high and fast compared with that of other long-term studies in central Europe, the study still showed that high amounts of nutrients remained. If the management applied on the experiment site were to be stopped or interrupted, we would expect that the weeds (*U*. *dioica and R*. *obtusifolius*) would emerge and become dominant once again. Therefore, removal of nutrients as well as eradication or suppression of *U*. *dioica and R*. *obtusifolius* with cutting management alone for some years will not be sufficient when the soil contains excess amounts of key nutrients.
4. Finally, considering the result from this experiment and other similar studies, we can see treatment with herbicide (glyphosate) application combined with cutting (2CH) did not demonstrate significant difference in removing nutrient from the soil/herbage compared to the nature friendly cut treatment (2C). We conclude restoration measures in national parks or other protected areas are better off without the application of destructive and non-selective herbicide as a potential measure against invasive weed species.

## Supporting information

S1 Fig Concentration of Ca (A), Cox (B), C:N (C), K (D), Mg (E), Total N (F), P (G) and pH/KCl (H), in the soil (10–20 cm). Error bars represent standard error of the means (SE). For treatment abbreviation (U, 2C, 2CH) see Table 1.
(DOCX)

S1 Table Botanical composition of semi-natural grassland species in the vicinity of the experiment. (DOCX)
