# Supplementary material for: Restoration management of cattle resting place in mountain grassland
Source: PLoS One. 2021 Apr 1;16(4):e0249445. doi: 10.1371/journal.pone.0249445 (PMC8016235; doi:10.1371/journal.pone.0249445)

**S1. Fig. Concentration of Ca (A), Cox (B), C:N (C), K (D), Mg (E), Total N (F), P (G) and pH/KCl (H), in the soil (10-20 cm). Error bars represent standard error of the means (SE). For treatment abbreviation (U, 2C, 2CH) see Table 1.**


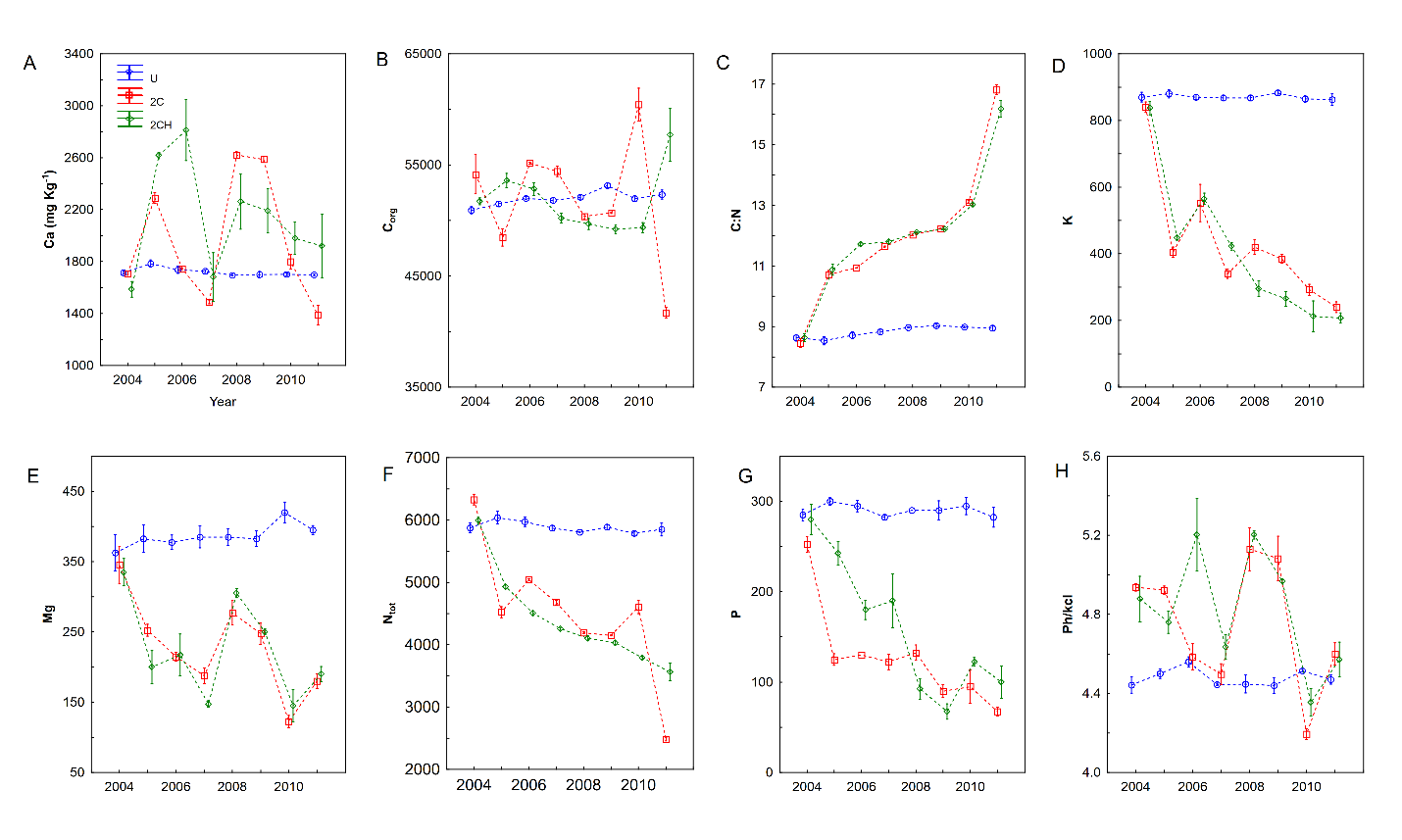

Supplement: S1 Fig — Concentration of Ca (A), Cox (B), C:N (C), K (D), Mg (E), Total N (F), P (G) and pH/KCl (H), in the soil (10–20 cm). Error bars represent standard error of the means (SE). For treatment abbreviation (U, 2C, 2CH) see Table 1. (DOCX) [file pone.0249445.s001.docx]
