# Supplementary material for: Restoration management of cattle resting place in mountain grassland
Source: PLoS One. 2021 Apr 1;16(4):e0249445. doi: 10.1371/journal.pone.0249445 (PMC8016235; doi:10.1371/journal.pone.0249445)
Supplement: S1 Table — (DOCX) [file pone.0249445.s002.docx]

**S1. Table. Table. Botanical composition of semi-natural grassland species in the vicinity of the experiment**

| **Species** | **2004** | **2005** | **2006** | **2007** | **2008** | **2009** | **2010** | **2011** |
| --- | --- | --- | --- | --- | --- | --- | --- | --- |
| *Agrostis capillaris* L. | 14 | 12 | 15 | 14 | 14 | 13 | 15 | 14 |
| *Briza media* L. | 2 | 3 | 2 | 2 | 2 | 3 | 2 | 2 |
| *Festuca rubra* L*.* ssp*. rubra agg.* | 51 | 53 | 50 | 51 | 52 | 52 | 51 | 51 |
| *Lotus corniculatus* L*.* | 2 | 2 | 2 | 2 | 2 | 3 | 1 | 2 |
| *Trifolium campestre* Schreb*.* | 0 | 0 | 0 | 0 | 0 | 1 | 0 | 0 |
| *Alchemilla vulgaris* L. | 7 | 6 | 6 | 5 | 6 | 6 | 8 | 6 |
| *Cruciata glabra* (L.) Ehrend. | 3 | 3 | 2 | 3 | 3 | 3 | 2 | 3 |
| *Galium verum* L*.* | 1 | 0 | 0 | 1 | 0 | 1 | 0 | 0 |
| *Leontodon autumnalis* L. | 5 | 5 | 5 | 6 | 4 | 6 | 4 | 5 |
| *Luzula luzuloides* (Lam.) Dandy et Wilmott | 1 | 1 | 1 | 1 | 2 | 1 | 1 | 0 |
| *Plantago lanceolata* L. | 0 | 1 | 0 | 0 | 0 | 0 | 0 | 0 |
| *Prunella vulgaris* L. | 2 | 2 | 2 | 2 | 2 | 2 | 1 | 2 |
| *Stellaria graminea* L. | 1 | 0 | 0 | 1 | 0 | 0 | 1 | 1 |
| *Thymus pulegioides* L. | 2 | 3 | 2 | 2 | 2 | 2 | 2 | 3 |
| *Veronica chamaedrys* L*.* | 0 | 0 | 0 | 0 | 0 | 0 | 0 | 1 |

**Reference**

Marchold, k., Hindak, F. Zoznam nizsich a vyssich rastlin Slovenska. Bratislava, veda, 1998, 687.
